# Matrix Metalloproteinases and Tissue Inhibitor of Metalloproteinases Are Essential for the Inflammatory Response in Cancer Cells

**DOI:** 10.1155/2010/985132

**Published:** 2010-07-20

**Authors:** Jun Sun

**Affiliations:** ^1^Gastroenterology & Hepatology Division, Department of Medicine, University of Rochester, Box 646, 601 Elmwood Avenue, Rochester, NY 14642, USA; ^2^Department of Microbiology and Immunology, University of Rochester, Box 646, 601 Elmwood Avenue, Rochester, NY 14642, USA; ^3^James Wilmot Cancer Center, University of Rochester, Box 646, 601 Elmwood Avenue, Rochester, NY 14642, USA

## Abstract

Inflammation plays a critical role in the development of cancer. Matrix Metalloproteinase (MMP) functions in the remodeling of the extracellular matrix that is integral for many normal and pathological processes such as morphogenesis, angiogenesis, tissue repair, and tumor invasion. The tissue inhibitor of metalloproteinases (TIMPs) family regulates the activity of multifunctional metalloproteinases. In this paper, we discuss the role and mechanism of MMP and TIMP in regulating inflammation responses in solid tumors. We discuss the mechanism of MMP and inflammation in melanoma, colon cancer, breast cancer, and prostate cancer. We highlight the roles of the TIMP-2 in modulating the proinflammatory NF-*κ*B pathway in melanoma and lung caner cells. Based on the molecular mechanisms of TIMPs and MMPs in inflammation and cancer, we can design new strategies for cancer therapy.

## 1. Introduction

MMPs belong to a family of structurally related proteolytic enzymes that mediate degradation of the extracellular matrix and the basement of membranes [1–3]. High levels of MMP activity have been linked to tumor growth, invasion, and angiogenesis inflammation and may even work in a nonproteolytic manner [4, 5]. The tissue inhibitor of metalloproteinases family, including TIMP-1, 2, 3, and 4, regulates the activity of multifunctional metalloproteinases [6, 7]. Among TIMP members, TIMP-2 is most frequently investigated because it is a unique member of the TIMP family and involved in cancer progression and metastasis. 

Recent studies have begun to unravel molecular pathways linking inflammation and cancer [8, 9]. Inflammatory conditions can initiate or promote oncogenic transformation and genetic and epigenetic changes in malignant cells. An inflammatory microenvironment further supports tumor progression [10–12]. Cancer-associated inflammation is marked by the presence of specific inflammatory cells and inflammatory mediators, including cytokines and chemokines. Nuclear factor-*κ*B (NF-*κ*B) transcription factor plays an essential role in innate and adaptive immune responses, cell proliferation, apoptosis, and tumorigenesis [13–15]. Constitutive activation of NF-*κ*B has been directly implicated in tumorigenesis of various cancer types [16–18]. Recent evidence also suggests a crucial role for signal transducer and activator of transcription (STAT) family in selectively inducing and maintaining a procarcinogenic inflammatory microenvironment, both at the initiation of malignant transformation and during cancer progression [11]. The targeting of inflammatory mediators (chemokines and cytokines, such as TNF-*α* and IL-1*β*), key transcription factors involved in inflammation (such as NF-*κ*B and STAT), or inflammatory cells decreases the incidence and spread of cancer [10]. Therefore, anti-inflammation is an essential strategy for the cancer therapy.

In this paper, we discuss the mechanisms of MMPs and TIMPs in regulating inflammation in the cancer cells. We highlighted the interaction among TIMPs, MMPs, and inflammatory pathways. We focus on the roles of MMP and TIMP-2 in modulating inflammation and cancer progression in solid tumors. We also discuss the progress on the therapeutic researches and lessons learned from the failed clinical trials for MMP inhibitors. Increasing studies demonstrate that chronic inflammation is associated with outcome of cancers [19]. Therefore, insights in the molecular mechanisms of MMP and TIMP in inflammation and cancer will provide promising opportunities for therapeutical intervention.

## 2. MMPs and TIMPs

The MMP family includes over 20 zinc-dependent enzymes that share common functional domains. These enzymes were initially characterized by their extensive ability to degrade extracellular matrix proteins including collagens, laminin, fibronectin, vitronectin, aggrecan, enactin, tenascin, elastin, and proteoglycans [20]. Recent studies further demonstrate that MMPs actually cleave many other types of peptides and proteins and have a myriad of other important functions independent of proteolytic activity [21]. 

The tissue inhibitors of metalloproteinases or TIMPs consist of a small family of four homologous and low molecular weight proteins. TIMPs suppress MMP activity critical for extracellular matrix turnover associated with both physiologic and pathologic tissue remodeling. TIMP concentrations generally far exceed the concentration of MMPs in tissue and extracellular fluids, thereby limiting their proteolytic activity to focal pericellular sites [22]. 

TIMPs, aside from inhibition MMP, also are involved in other biological process required for metastasis and angiogenesis [23]. TIMP-2, a unique member of this family, was discovered in 1989 [24]. TIMP-2 selectively blocked human microvascular endothelial cell growth in vitro in response to proangiogenic factors such as fibroblast growth factor 2(FGF-2) or vascular endothelial growth factor A (VEGF A) [25]. Other distinguishing features of TIMP-2 are that it is the only member that is not nested within the gene structure of the synapsin gene family and the *timp-2* gene also contains a large first intron (>60 kB). TIMP-2 could suppress receptor tyrosine kinase signaling independent of metalloproteinase inhibition [25]. TIMP-2 bridges the interaction between the MMP-2 zymogen and MT1-MMP. In contrast to the usual inhibitory role, at low TIMP-2 concentration, an adjacent TIMP-2-free MT1-MMP can effectively activate proMMP-2. However, at high TIMP-2 concentration, all of the cell surface MT1-MMP undergoes complex formation with TIMP-2, thereby inhibiting proMMP-2 activation [26]. The cellular mechanism for the control of MT1-MMP catalytic activity involved concurrent reciprocal modulation of TIMP-2 expression by ERK1/2 and p38 MAPKs. Inhibition of ERK1/2 phosphorylation decreased TIMP-2 production, and downregulation of p38 MAPK activity enhanced TIMP-2 synthesis [26]. Recently, TIMP-1 is shown to bind to CD63, thus regulating cell survival and polarization [27]. TIMP3 is also assigned a new function independent of its MMP-inhibitory activity [28]. TIMP3 blocks the binding of VEGF to VEGF receptor-2 and inhibits downstream signaling and angiogenesis [28].

## 3. General Roles of MMPs and TIMPs in Cancers

Tumor cells produce enzymes that destroy the matrix barriers surrounding the tumor, permitting invasion into surrounding connective tissues, entry and exit from blood vessels, and metastasis to distant organs. Enzymes that degrade the extracellular matrix (ECM) have long been viewed as essential for tumor progression. MMPs are able to degrade virtually all ECM components. Therefore, classically, MMPs were recognized as being produced and secreted by tumor cells, degrading basement membrane and extracellular matrix components, thereby facilitating tumor cell invasion and metastasis. Now, we know that MMPs are frequently produced by surrounding stromal cells, including fibroblasts and infiltrating inflammatory cells. One explanation for this phenomenon is that cancer cells produce Extracellular Matrix Metalloproteinase Inducer, a cell surface glycoprotein, which directly stimulates fibroblasts to produce MMP1, 2, 3, and MMP14 [29]. 

Recent studies further demonstrate that MMPs contribute to multiple steps of tumor progression in addition to invasion, including tumor promotion, angiogenesis, and the establishment and growth of metastatic lesions in distant organ sites. MMPs are upregulated in virtually all human and animal tumors as well as in most tumor cell lines [20, 30]. The stage of tumor progression is positively correlated with the expression of MMP family members (MMP-1/interstitial collagenase; MMPs 2, 3, 7, 9, 11, and 14) [21]. Moreover, MMPs also solubilize cell surface and matrix-bound factors that can then act in an autocrine or paracrine manner to influence cellular properties such as growth, death, and migration. Changes in MMP levels can markedly affect the invasive behavior of tumor cells and their ability to metastasize in experimental animal models [31]. Taken together, these studies suggest that MMPs are important contributors to tumor progression. 

TIMPs are originally known to inhibit the MMP activities. TIMP is downregulated or silenced in a variety of human cancer cell lines. TIMPs also are involved in other biological processes in cancer. Promoter hypermethylation and lost expression of *TIMP-2* gene have been reported in prostate cells and tumor samples [32]. Overexpression of TIMPs reduced experimental metastasis of melanoma [33, 34]. Intraperitoneal injection of recombinant TIMP-1 is explored in lung cancer [35]. Transgenic studies reveal that mouse 3T3 cells became tumorigenic after antisense depletion of TIMP-1 [36]. TIMP-1 overproduction slowed chemical carcinogenesis in skin and liver carcinogenesis in transgenic mice [37, 38]. In addition, TIMPs have shown apoptosis-inducing properties (TIMP3). Overexpression of TIMP-3 resulted in apoptosis of A549 lung cancer cells and AdCMVTIMP3 up-regulated the expression of p53, Fas ligand, TNFR1, and TNFR2 on these cells. Adenoviral delivery of TIMP-3 gene inhibited the growth of pre-established A549 tumours in Balb/c nude mice, and was associated with a greater therapeutic effect than either TIMP-1 or -2 gene delivery. These findings establish the potential of adenoviral gene delivery of TIMP3 as a therapeutic agent for selected lung cancers [39]. Study using breast cancer samples demonstrates that the expressions of MMP-1, MMP-2, MMP-3, MMP-9, urokinase-type PA, and tissue-type PA, and inhibitors (TIMP-1 and TIMP-2) were stronger or equivalent in tumor cells than in fibroblasts or inflammatory cells within the tumor section [40]. Overall, studies on MMPs and TIMPS in cancer provide the rationale for developing cancer drugs that target TIMP and MMP activities.

## 4. MMPs and Cytokines

The activity of MMP is under tight control at several levels* in vivo*: transcriptionally by growth factors, hormones, cytokines, and oncogenes; posttranscriptionally by alteration of mRNA stability; and at the enzymatic activity level by activation of the latent form and inhibition by TIMPs [23]. 

MMPs are generally expressed in very low amounts and their transcription is regulated either positively or negatively by cytokines and growth factors such as inflammatory interleukins (IL-1, IL-4, IL-6, TNF) or transforming growth factors (EGF, HGF, TGF*β*) [41]. The importance of cytokines such as TNF-*α*, interleukin (IL)-1, and IL-6 in stimulating production of MMPs in disease has been emphasized. They can be seen as important therapeutic targets for intervention in cancers. Increasing evidences demonstrate that MMPs play an important role in acute as well as chronic inflammation [5, 42]. CCL8/MCP-2 is processed by MMP-1 and MMP-3. The proteolytic cleavage of CCL8 can counteract the antitumor capacity of this chemokine in a melanoma model [43]. The proteolytic cleavage of a chemokine by MMPs can have great impact in a clinically relevant setting of tumor development [5].

The transcription of TIMPs is regulated by similar cytokines and growth factors that control MMP expression, that is, TGF*β*, TNF*α*, IL-1, and IL-6 [44]. However, in macrophages IL-4 and IFN gamma released by lymphocytes suppress metalloproteinase biosynthesis without affecting TIMP production [45, 46]. 

IL-10 is a cytokine with potent anti-inflammatory properties, repressing the expression of inflammatory cytokines such as TNF-alpha, IL-6, and IL-1. IL-10 enhances TIMP-1 production while decreasing metalloproteinase biosynthesis in tissue macrophages, and blood monocytes TIMP-2 production was not affected. IL-10 regulation was cell type specific, as it had no effect on the production of metalloproteinases or TIMP by human fibroblasts [47]. In nonimmortalized primary human prostate cell strains, IL-10 activation of the IL-10 receptor blocked MMP-2 and membrane type 1 (MT1)-MMP transcription and protein synthesis. IL-10 induced protein(s) binding to a putative “silencer element” downstream of the p53 binding site. The data show that IL-10 blocks IGF-I activation of MMP-2 and MT1-MMP mRNA expression and protein synthesis in prostate cells [48]. In summary, IL-10 has a potent and unique effect by enhancing TIMP production while decreasing metalloproteinase biosynthesis in cells.

## 5. TIMP-2 Directly Modulates the NF-*κ*B Pathway Activity

NF-*κ*B is an inducible dimeric transcription factor composed of the RelA (p65) and NF-*κ*B1 (p50) subunits [49]. NF-*κ*B activation involves its release from its inhibitor, I*κ*B*α*, and its subsequent translocation from the cytoplasm to the nucleus, where it binds to promoters of target genes. Many proinflammatory cytokines and chemokines, such as IL-8, IL-6, and TNF, are targets of NF-*κ*B regulation [50, 51]. Using stable melanoma cell lines, parental A2058, A2058T2-1 overexpressing TIMP-2, and A2058T2R-7 underexpressing TIMP-2, we demonstrate that the IL-8 secretion and IL-8 mRNA expression significantly increased in the A2058T2-1 overexpressing TIMP-2 [52]. We also found that in the TIMP-2 overexpressed cells the basal level of I*κ*B*α* was lower than that in the parental A2058 cells. I*κ*B*α* degradation involves phosphorylation, ubiquitination, and subsequent proteasomal degradation. Phospho-I*κ*B*α* was increased in the TIMP-2 over-expressed cells, which is consistent with the reduced level of total I*κ*B*α*. TIMP-2 expression is able to elevate p65 phosphorylation, thus increasing the NF-*κ*B activity (total I*κ*B*α*↓ =p-I*κ*B  *α*↑ = p-p65↑ = activity of NF-*κ*B↑). TIMP-2 expression directly upregulates the transcriptional activity of NF-*κ*B [52].

Apoptosis is one of the biological effects regulated by the NF-*κ*B pathway. TIMP-2 overexpression is able to protect cells from apoptosis. TIMP-2 was shown to stimulate proliferation in human cells, including osteosarcoma cells [53], fibroblasts [54], and A549 lung adenocarcinoma cells [55]. Our data suggest that TIMP-2 over-expression is able to protect cells from apoptosis in human melanoma A2058 cells [52]. It is consistent with the previous studies that TIMP-2 overexpression protects B16F10 melanoma cells from apoptosis reduced [23]. Overall, these data indicate that TIPM-2 modulates other relevant aspects of the melanomatatic phenotypes including cell proliferation and cell survival. 

In lung cancer cells, the NF-*κ*B activity was increased by exposure to TIMP-2 [56]. The NF-*κ*B transcription factor is known to act as a tumor promoter. It is intriguing that TIMP-2 upregulates NF-*κ*B activity, whereas TIMP-2-overexpression can prevent tumor invasion [23]. TIMP-2 upregulation of NF-*κ*B activity may inhibit tumor growth in the early stage because other data suggest a dual function of NF-*κ*B during tumor progression. In the early stages, NF-*κ*B inhibits tumor growth; as further mutations lead to a loss of tumor suppressor expression, the oncogenic functions of NF-*κ*B become unleashed, allowing it to actively contribute to tumorigenesis [57]. 

**Figure 1:**
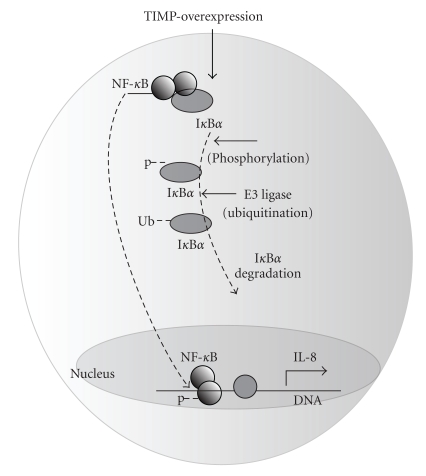
TIMP-2 expression and NF-*κ*B activity. The effects of TIMP-2 on the NF-*κ*B pathway include the decreased basal level of I*κ*B*α*, increased phosphorylation of I*κ*B*α* and NF-*κ*B, increased transcriptional NF-*κ*B activity, and elevated IL-8 levels in the TIMP-2-overexpressed cells.

Overall, TIMP-2 expression can directly modulate the NF-*κ*B pathway (Figure 1). The effects of TIMP-2 on the NF-*κ*B pathway include the decreased basal level of I*κ*B*α*, increased phosphorylation of I*κ*B*α* and NF-*κ*B, increased transcriptional NF-*κ*B activity, and elevated IL-8 levels in the TIMP-2-overexpressed A2028T2-1 cells. Consequently, TIMP-2 over-expression was able to protect cells from apoptosis [52]. Our results and other's publications show that TIMP-2 over-expression is sufficient to increase the NF-*κ*B activity and protect cells from apoptosis. These data emphasize the critical role of TIMP-2 in modulating cell survival and invasion through the NF-*κ*B activity.

## 6. STAT, MMPs, and TIMPs

The signal transducers and activators of transcription (STATs) are members of a ubiquitously expressed family of transcription factors activated in response to growth factors and cytokines. STAT3 has been shown to be an oncogene [58]. Many types of human cancers express constitutively active STAT3 [58]. Increasing evidences demonstrate that STAT regulates MMPs [59]. STAT3 up-regulates TIMP-1 in certain cell lines. For example, A significant association exists between the expression of the phosphorylated/active form of STAT3 (pSTAT3) and that of TIMP1. Importantly, STAT3 activation correlated significantly with a lower frequency of vascular and lymphatic invasion. STAT3 activation may modulate tumor invasiveness of breast cancer by regulating TIMP1 expression [60].

TIMP-1 gene is up-regulated by IL-6 [61]. A molecular biology study identified that TIMP-1 is a downstream target of STAT3. *Timp-1* gene possesses an IL-6/oncostatin M (OSM) response element. Within this element, there are two functional binding sites for transcription factors activator protein-1 (AP-1) and STAT. IL-6/OSM stimulation induces binding of STAT3 to the IL-6/OSM response element, while binding of the AP-1 protein was constitutive. Binding sites for both AP-1 and STAT3 are necessary for full responsiveness of the TIMP-1 promoter to IL-6/OSM, as shown by deletion and mutation analysis. Furthermore, the entire IL-6/OSM response element conferred responsiveness onto a heterologous promoter [61]. As a downstream target of STAT3, TIMP1 mediates the antiapoptotic effects of STAT3 [62].

The IL-10 receptor is in the JAK/STAT class of receptors [63]. IL-10 blocked MMP-2 and MT1-MMP transcription and protein synthesis in an IL-10 receptor-dependent manner [48]. Therefore, it will be interesting to investigate whether the regulation of IL-10 on TIMP and MMP is through the JAK/STAT pathway.

## 7. Conclusion and Future Studies

Based on the important role of MMPs cancer and successful drug trials in mice, numerous clinical trials were initiated in the 1990s to test the effectiveness of hydroxamic acid-derived MMP inhibitors (MMPIs) in patients with cancer. However, these drugs have languished. Retrospective assessment of the design of clinical trials has led to the recognition that specific MMPIs used in conjunction with cytotoxic chemotherapy in early stage, rather than late stage cancer, needs future consideration [31]. The important lessons learned from the MPI experience may be of great value for future studies of MPIs and for cancer drug development in general [31]. 

Eempiric evidence for control and modulation of MMP transcription and/or activation by several naturally occurring substances, such as flavonoids, green tea polyphenols, and curcumin, represent novel options for the control of MMP activity even in early tumor stages. Additionally, these substances have little or no toxic side effects and good bioavailability, and therefore their continuing analysis provides intriguing insight into tumor pathophysiology and possibly new therapeutic options [64].

Increasing evidences demonstrate that TIMPs could modulate critical signaling pathways independent of metalloproteinase inhibition. These findings suggest that further understanding of the antiangiogenic activity of TIMPs might be exploited in human cancer therapy.

Cytokine and chemokines display diverse effects for enhancing immunity to tumor-associated antigens, regulating angiogenesis, promoting proliferation/antiapoptosis of tumor cells, and mediating tumor cell invasion and trafficking in an organ-specific manner that leads to metastases. They can be seen as important therapeutic targets for intervention in cancer [65].

**Figure 2:**
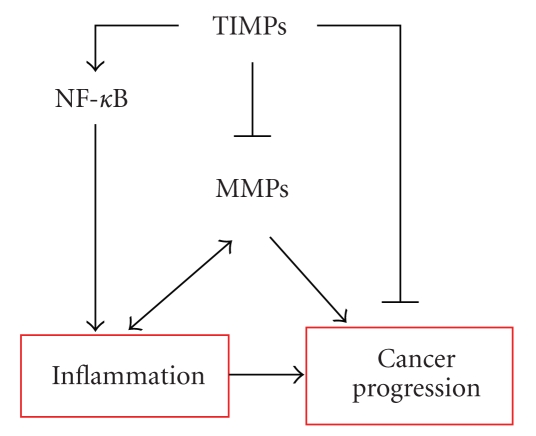
TIMPs and MMPs in inflammation and tumor progression. TIMPs functions in inhibition of MMP activity, activation of the proinflammatory NF-*κ*B pathway, and regulation of inflammation. MMPs play a critical role in tumor progression. Inflammatory cytokines enhance the dysfunction of MMPs, whereas MMPs increase inflammation in the tissue. TIMPs may modulate critical signaling pathways for inflammation and cancer independent of metalloproteinase inhibition.

Cancer progression is known to be involved in the NF-*κ*B and STAT pathways. NF-*κ*B is an important target to prevent metastasis and provide a rationale for further study of this transcription factor in metastatic disease [66]. TIMPs functions in inhibition of MMP activity, activation of the proinflammatory NF-*κ*B pathway, and regulation of inflammation. MMPs play a critical role in tumor progression. Inflammatory cytokines enhance the dysfunction of MMPs, whereas MMPs increase inflammation in the tissue (Figure 2). Therefore, understanding inflammation regulated by MMP and TIMP will provide the platform for the design of therapies. The therapeutic implications of anti-inflammation and anticancer will be an exciting and promising field of translational studies.
